# Perspectives, Knowledge, and Fears of Cancer Patients About COVID-19

**DOI:** 10.3389/fonc.2020.01553

**Published:** 2020-08-28

**Authors:** Deniz Can Guven, Taha Koray Sahin, Oktay Halit Aktepe, Hasan Cagri Yildirim, Sercan Aksoy, Saadettin Kilickap

**Affiliations:** Department of Medical Oncology, Hacettepe University Cancer Institute, Ankara, Turkey

**Keywords:** coronavirus disease 2019 (COVID-19), pandemic, oncologic care, patient education, questionnaire, coronavirus disease 2019 (COVID-19) fear, coronavirus disease 2019 (COVID-19) knowledge

## Abstract

Coronavirus disease 2019 (COVID-19) is expected to significantly affect cancer patients due to adverse outcomes with COVID-19 and disruptions in cancer care. Another important point is the stress and anxiety burden of COVID-19, which could affect quality of life. Patient education is vital due to the vulnerability of the topic to disinformation. To determine the areas needing improvements in patient education, and coping with stress, the burden of the problem should be pictured. From this point, we aimed to assess the perspectives and fears of cancer patients about COVID-19 with resources of COVID-19 knowledge with a questionnaire. A total of 250 adult cancer patients applied to the outpatient chemotherapy unit of Hacettepe University Cancer Center between May 27, 2020, and June 9, 2020, invited to answer a questionnaire of 13 multiple-choice questions with a return rate of 78% (195/250). Most patients acquired their knowledge about COVID-19 from television (91.9%). Social media were the second most common source of knowledge (43.8%) with a predilection in younger patients, nonsmokers, targeted therapy- or immunotherapy-treated patients, and breast cancer patients (>65 vs. <65 years of age, *p* = 0.057; nonsmoker vs. ever-smoker, *p* = 0.036; targeted therapy and immunotherapy vs. chemotherapy, *p* = 0.004; breast cancer vs. other cancers, *p* = 0.019). The percentage of patients seeing the information about COVID-19 as adequate (38.9%) or inadequate (35.1%) was similar. More than 90% of the patients had a moderate to severe degree of COVID-19 fear. In addition, 27.6% of patients had false knowledge of glove using as a protective measure for COVID-19. More than half of the patients had another wrong knowledge as the need for the supplements for COVID-19 protection. A significant percentage of patients (84.7%) expected some level of disruption in oncological care with the expectation of a moderate-to-severe disruption was more common in the advanced-stage patients (*p* = 0.026). In our experience, most cancer patients had a significant degree of fear about both infecting COVID-19 and the disruption of cancer care by COVID-19. A significant amount of our patients had wrong information about the protection necessities, which denotes the need for better patient education about COVID-19.

## Introduction

In December 2019, a group of viral pneumonia cases secondary to a novel coronavirus was reported from Wuhan, China (1). The disease was first regarded as a zoonotic infection; however, human-to-human transmission was demonstrated after a short notice (2). The clinical disease was named the coronavirus disease 2019 (COVID-19) (3) and declared a pandemic by the WHO in March 2020. In a matter of weeks, COVID-19 spread rapidly to all parts of the world (4).

The COVID-19 stormed the world in the last 5 months with significant damage to both infected patients and health care in general due to the state of paralysis in health care while dealing with the burden created by COVID-19 (5). The general mortality of the disease was around 2–10% with an increased risk of mortality and poor prognosis in elderly patients and patients with chronic diseases including cancer. Oncologic patients were expected to be affected by the pandemic due to an increased risk of adverse outcomes in these patients (6, 7). There is also a risk of interruptions in optimal cancer care during the pandemic, as pointed out by several oncology societies (8–10). Another important but rather less talked about point is the stress and anxiety burden of COVID-19 in cancer patients, which could greatly affect quality of life.

The knowledge about COVID-19 is ever-expanding. However, due to the lack of robust scientific evidence, the topic is very vulnerable to disinformation (11, 12), which can lead to risky behaviors by patients. Health authorities and organizations published recommendations for patient information and education, although their dissemination and implementation in daily life are variable. For the better determination of the areas needing improvements in patient education, and coping with stress, the burden of the problem should be pictured. To date, only a few studies addressed the knowledge, perspectives, and fears of cancer patients about the COVID-19 pandemic and the pandemic effects on patient care (11, 12). From this point, we aimed to assess the perspectives and fears of cancer patients about COVID-19 with levels and resources of COVID-19 knowledge with a questionnaire.

## Methods

### Patients and Questionnaire Development

Adult cancer patients applied to the outpatient infusional chemotherapy unit of Hacettepe University Cancer Center between the May 27, 2020, and June 9, 2020, invited to answer the questionnaire. The questionnaire was developed for the present study and applied in person during the applications for chemotherapy. It consisted of 13 multiple-choice questions, including questions about the baseline disease and patient characteristics, smoking habits, sources of the COVID-19 knowledge, the grade of COVID-19 fear, changes in the daily habits during COVID-19, and perspectives about the effects of COVID-19 to cancer care. Most questions about patients' perspectives and fears included a grading system with grades spanning from 1 to 5. With most questions, a three-category reclassification was conducted for answers with scores of 1 and 2 regarded as mild, a score of 3 as moderate, and scores of 4 and 5 were severe levels (Appendix).

### Statistical Analyses and Ethical Approval

Patient demographics and tumor types were recorded to a database with questionnaire answers. Baseline characteristics (age, sex, and disease characteristics) and answers were expressed by the descriptive statistics (medians, frequencies, and percentages). Chi-square and Fisher exact tests were used to compare the categorical variables. *P*-values below 0.05 were considered statistically significant. Statistical Package for Social Sciences version 26 program was used in the analyses. All procedures in the study followed the ethical standards of the institutional research committee as well as the 1964 Helsinki declaration and its later amendments. The study was approved by the local ethics committee and the Republic of Turkey Ministry of Health.

## Results

**Figure 1:**
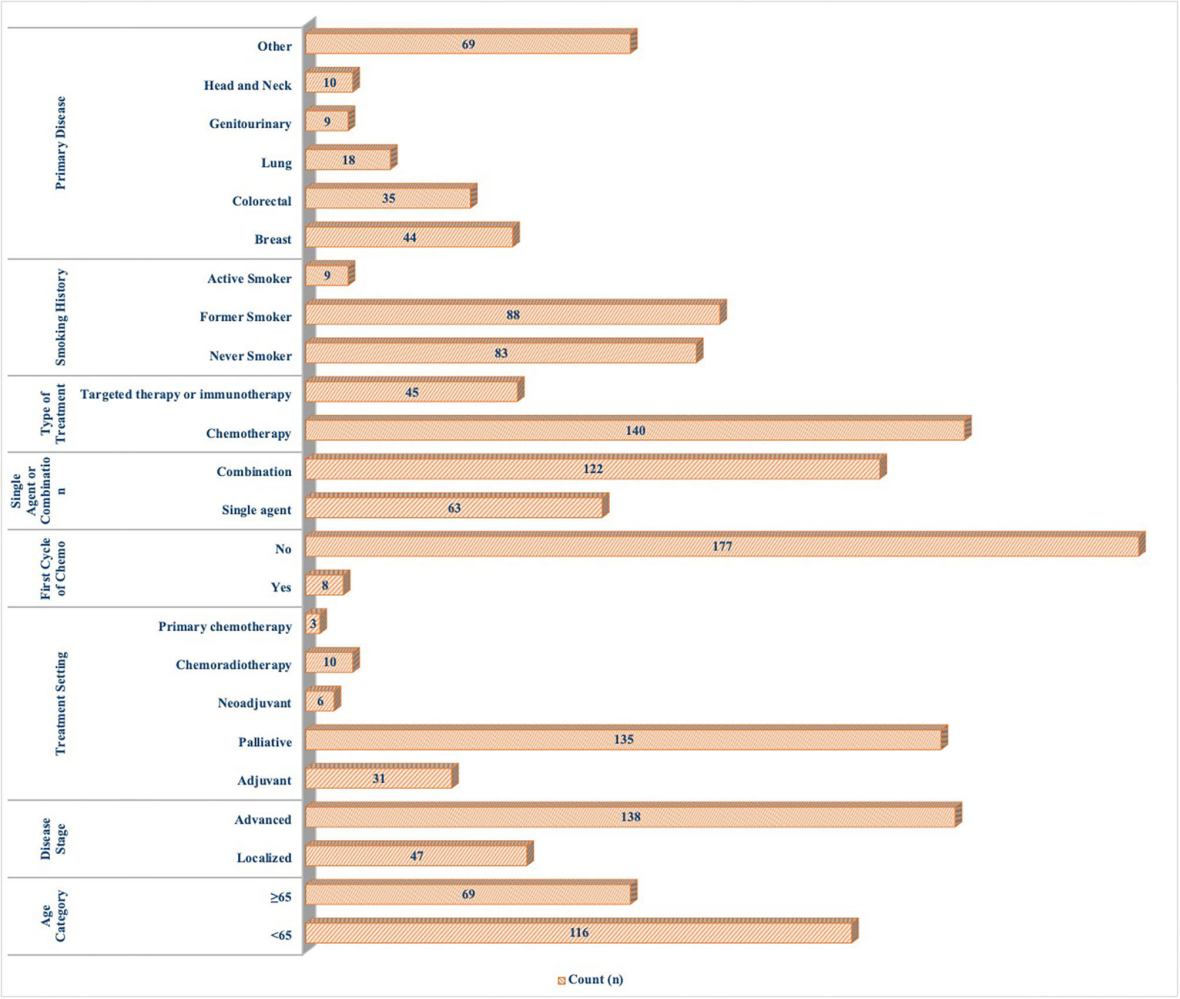
Baseline characteristics of patients.

A total of 195 questionnaires were collected with a return rate of 78% (195/250). The median age of our cohort was 59 (20–82) years, and 57% of the patients were females. More than a third of patients were over 65 years of age. Most patients were under palliative treatment for the advanced-stage disease (74.6%). Similar to the incidences in the general population, breast cancer was the most frequent diagnosis (23.8%), followed by colorectal cancer (18.9%) and lung cancer (9.7%). More than half of the patients had a smoking history (53.9%; Figure 1).

Most patients acquired their knowledge about COVID-19 from television (91.9%), with all geriatric patients reporting television use as a source of information about COVID-19 (>65 vs. <65 years of age, *p* = 0.002). Social media were the second most common source of knowledge (43.8%) with a predilection in younger patients, nonsmokers, targeted therapy- or immunotherapy-treated patients, and breast cancer patients (>65 vs. <65 years of age, *p* = 0.057; nonsmoker vs. ever-smoker, *p* = 0.036; targeted therapy and immunotherapy vs. chemotherapy, *p* = 0.004; breast cancer vs. other cancers, *p* = 0.019). The percentage of patients seeing the information about COVID-19 as adequate (38.9%) or inadequate (35.1%) was similar. More male patients stated that their knowledge about the COVID-19 was inadequate compared to female patients (*p* = 0.001). More than 90% of the patients had a moderate to severe degree of COVID-19 fear. While surgical or N95 masks were chosen as a necessity for cancer patients during hospital visits by all patients, 27.6% of patients had the false knowledge of gloves using as a protective measure for COVID-19. The percentage of patients with the knowledge of gloves as a necessity for protection did not differ with age and gender (>65 vs. <65 years of age, *p* = 0.395, male vs. female, *p* = 0.512). More than a quarter (28.6%) of patients viewed face shields among the necessary equipment for oncology patients. More than half of the patients also had another wrong knowledge as the need for supplements for protection from COVID-19.

**Figure 2:**
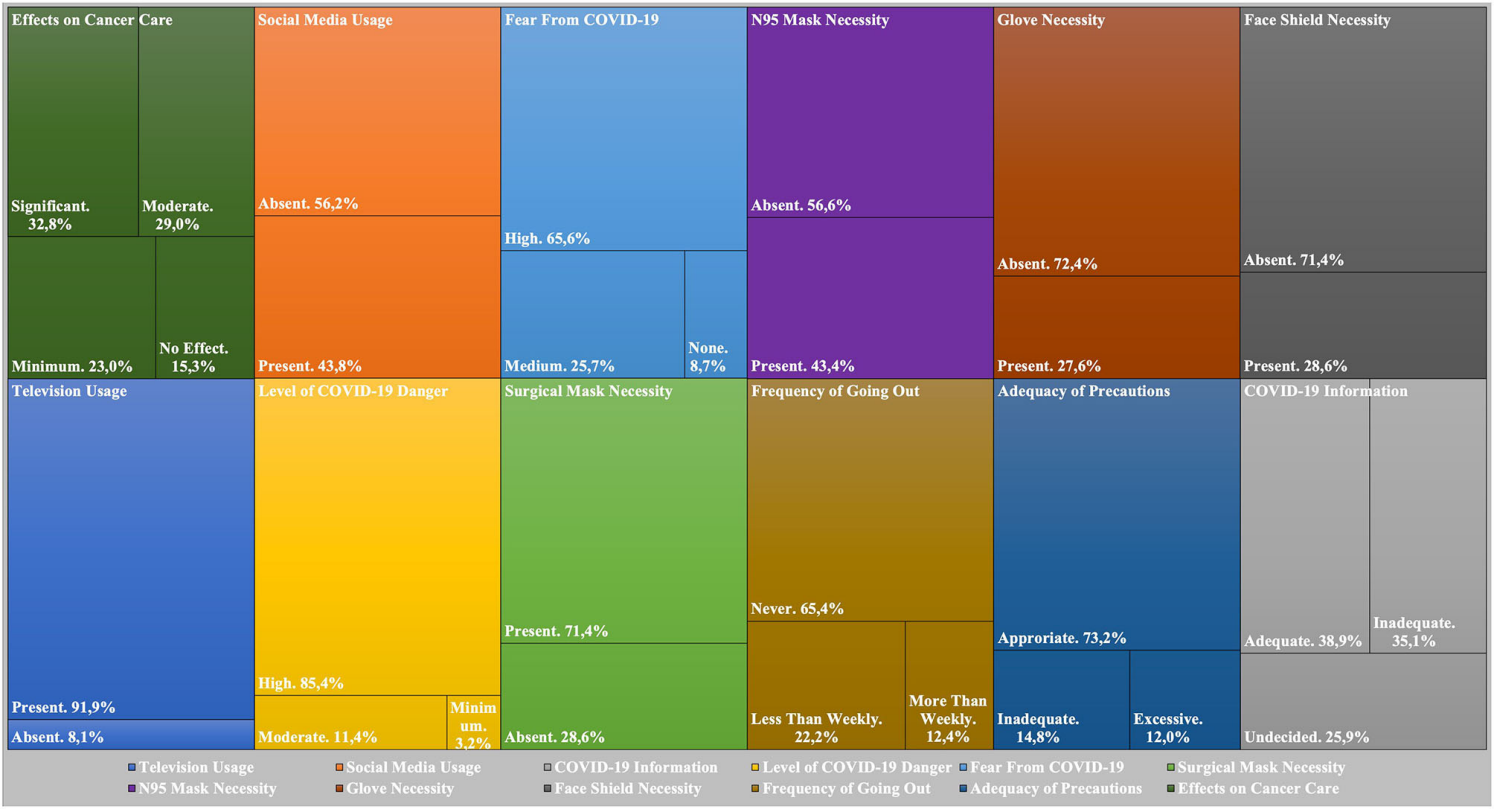
Answers of patients to survey questions.

Most patients saw treating oncologists at least once during the pandemic, mostly in the hospital. In addition, 73.2% of the patients stated they considered the institutional precautions sufficient, while some patients stated the need for further precautions or stated complaints about the excessive nature of the precautions. A higher percentage of patients (70%) treated with chemotherapy were completely isolated in their homes compared to the patients treated with targeted therapy or immunotherapy (51.1%; *p* = 0.020). Compatible with the low number of cases in the city of our hospital, only a few patients (3/185) had a known relative positive for COVID-19. Some patients changed their homes (13/185) during the pandemic for COVID-19 protection. The habitation changes were less frequent in patients treated with palliative intent (*p* < 0.001) possibly due to a need for caregivers in these patients. A significant percentage of patients (84.7%) expected some level of disruption in oncological care due to the COVID-19 pandemic. While the expectation of a moderate-to-severe disruption was similar in the different ages and genders (>65 vs. <65 years of age, *p* = 0.103, male vs. female, *p* = 0.165), it was more common in the advanced-stage patients (*p* = 0.026; Figure 2).

## Discussion

In this study, we evaluated the perspectives, knowledge sources, and fears of cancer patients about COVID-19. We found that almost all of our patients had some degree of COVID-19 fear, and more than 80% of patients expected disruptions in cancer care. There were differences in the sources of knowledge among patients, and a significant portion of patients had false knowledge about COVID-19.

COVID-19 arguably created the most significant pandemic in a century (13). With the increased globalization, the disease rapidly spread to all five continents (14). Both the high mortality and morbidity of disease (15) and the necessity of strict isolation measures and lockdowns (16) created fear and anxiety in society. There is an effort to quantify the stress and anxiety of COVID-19 by questionnaires in the general population. Consequently, a brief mental health screener (17) and Fear of COVID-19 Scale (18) were developed and validated in the general population. However, there are only two studies specifically evaluating cancer patients' fear and anxiety. Casanova et al. (11) evaluated the fear and views of the young cancer patients (14–21 years of age) about COVID-19 with a qualitative survey. A significant degree of fear and also discontent with the information from the media were reported similar to our study (11). In another study by Gebbia et al. (12), the WhatsApp Messenger conversations of 446 cancer patients were evaluated for the patients' needs and fears together with taken actions. In this study, a significant proportion of the cancer patients had a significant degree of fear and negative thoughts similar to our study. Unlike our study, the fear of COVID-19 contagion was much more significant than the concern about cancer care, as suggested by the request of postponing visits and even adjuvant treatments by the patients (12). We think that this difference is due to the higher COVID-19 burden and prevalence in Sicily, Italy, compared to Turkey during the study period. There is a significant risk of nosocomial contamination in the areas which are significantly affected by COVID-19, as evidenced by the more than a 1/3 in hospital contamination in the first series (19) and a 28% nosocomial contamination in the inpatient data of cancer patients from Wuhan, China (20). So, the fear of infection is expected to be higher in these COVID-19 red zones.

Although there are significant improvements and the progress about COVID-19 is astonishing with the help of collaboration of the scientific society, however, the topic is very vulnerable to contamination and poor scientific evidence possibly due to research exceptionalism (21). Also, there is info pollution in social media and televisions (22), which could lead to wrong health behaviors as evidenced by the high rate of glove and supplement use thoughts among our patients. A significant number of patients complained about the insufficient knowledge of COVID-19. These points should be considered by health authorities going forward.

Our study has several limitations. First of all, we did not use a previously validated questionnaire due to the lack of questionnaires validated in the Turkish language during our study period. The lack of education levels and marital statuses made us unable to make analyses according to theses factors. Another important point is the questions about the generalizability of our findings to the patient population of smaller community hospitals and also the hospitals in cities that are significantly affected by the pandemic. In these settings, the COVID-19 fear could be higher. The main strength of our study is the evaluation of both infecting with COVID-19 and having an impaired cancer care fear together with the sources of patient knowledge and the accuracy of the general knowledge about COVID-19 in a multidimensional manner. The study was conducted in a rather large patient sample and included patients with a wide range of ages and patients with a variety of tumors rather than a specific age or tumor group (11, 23).

## Conclusion

In our experience, most of the cancer patients had a significant degree of fear about both infecting with COVID-19 and also the disruption by COVID-19 in cancer care. A significant amount of our patients had the wrong information about protection necessities and discontent about the adequacy of information, which denote the need for better patient education about COVID-19.

## Data Availability Statement

The raw data supporting the conclusions of this article will be made available by the authors, without undue reservation.

## Ethics Statement

The studies involving human participants were reviewed and approved by Ethics Committee of Hacettepe University. The patients/participants provided their written informed consent to participate in this study.

## Author Contributions

DG and SK planned the work. DG, TS, OA, HY, SA, and SK participated in patient care and data collection. All authors, namely DG, TS, OA, HY, SA, and SK made significant and substantive contributions to the reporting of the work. All authors participated in the review of relevant literature, drafting of the manuscript, review, and revisions of the final draft. DG, TS, SA, and SK analyzed the data and determined the main conclusions. DG prepared the first draft of the manuscript. All authors reviewed and participated in the preparation of the revised and final version of the manuscript. DG and SK are responsible for the overall content as guarantors. All authors contributed to the article and approved the submitted version.

## Conflict of Interest

The authors declare that the research was conducted in the absence of any commercial or financial relationships that could be construed as a potential conflict of interest.
