# Supplementary material for: Perspectives, Knowledge, and Fears of Cancer Patients About COVID-19
Source: Front Oncol. 2020 Aug 28;10:1553. doi: 10.3389/fonc.2020.01553 (PMC7493662; doi:10.3389/fonc.2020.01553)
Supplement: Supplementary file 1 [file Data_Sheet_1.PDF]

Appendix: Perspectives and Fear of Cancer Patients about COVID-19 Questionnaire.

Patient Age: Gender: □ Female □ Male Primary Disease Diagnosis:

Smoking Status: □ I never smoked □ I quit □ I still smoke

Question-1: Where do you usually get information about coronavirus? (You can choose more than one option.)

□ Television □ Social media □ Health ministry statements

□ Households □ Other (Please explain):…

Question-2: Do you think that adequate information is provided in the media about the coronavirus? (Your rating is from 1 to 5. "1-I definitely do not think the provided information is adequate. 5-I'm definitely think the provided information is adequate.)

□ 1 □ 2 □ 3 □ 4 □ 5

Question-3: To what extent do you consider the coronavirus epidemic dangerous?

□Very low level □ Low level □ Medium level □ High level □ Very high level

Question 4: How afraid are you getting Coronavirus? (Your rating is from 1 to 5. "1 is not afraid at all-5 is extremely afraid")

□ 1 □ 2 □ 3 □ 4 □ 5

Question-5: Did you talk to your oncologist about what you should pay attention to during the coronavirus epidemic?

□ I never spoke □ I spoke once □ I spoke more than once

Question-6: Which channel did you use for reaching your oncologist during the coronavirus pandemic? (You can choose more than one option.)

 Face to face in the hospital Via phone Via e-mail

Question-7: What protective materials do you think cancer patients should use during the Coronavirus pandemic when applying to the hospital? (You can choose more than one option.)

 Surgical mask N95 Mask Glove Face Shield Other (explain): ……

Question-8: Do you think that cancer patients should consume any supplement for protection during the coronavirus epidemic?

 No Yes (explain): ………

Question-9: During the coronavirus pandemic, how often do you go outside, other than coming to the hospital?

Every day Several days a week Once a week Less than once a week Never

Question-10: Has anyone in your neighborhood been diagnosed with Coronavirus?

 No Yes (indicate your proximity): …………………

Question-11: During the Coronavirus pandemic, did you need to change your home or move someone from your home to reduce contact?

 Yes No

Question-12: How would you rate the adequacy of the measures taken in our hospital during the coronavirus epidemic period?

☐Excessively inadequate ☐ Somewhat insufficient ☐ Adequate

☐ Somewhat excessive ☐ Excessively excessive

Question-13: To what extent do you think the coronavirus epidemic will affect the oncology treatments and follow-ups? (Your rating is from 1 to 5. "1 is it will not affect at all and 5 is it will affect it excessively.")

☐ 1 ☐ 2 ☐ 3 ☐ 4 ☐ 5
